# Disparities in the Access to Different Types of Long-Term Care Services in Taiwan

**DOI:** 10.1093/geroni/igaa057.2477

**Published:** 2020-12-16

**Authors:** Ya-Mei Chen, Yuchi Young, Shih-Cyuan Wu, Hsiu-Hsi Chen, Yen-Po Yeh, Wan-Yu Chiu, Kuo-Piao Chung

**Affiliations:** 1 National Taiwan University, Taipei, Taiwan (Republic of China); 2 SUNY at Albany, Rensselaer, New York, United States; 3 National Taiwan University, Taipei, Taipei, Taiwan; 4 Changhua County Public Health Bureau, Changhua, Taiwan (Republic of China); 5 Institute of Health Policy and Management, National Taiwan University, Taipei, Taipei, Taiwan

## Abstract

Taiwan implemented version 1.0 of its 10-Year Long-Term Care Plan (TLTCP 1.0) in 2007, and version 2.0 (TLTCP 2.0) in 2017. The aim was to develop a system of home- and community-based services (HCBS) for long-term care (LTC). This study assessed and compared disparities in access to LTC services using one county’s database that claims made by 14,051 older adults who applied for LTC services from TLTCP 1.0 (n = 5,025) and TLTCP 2.0 (n = 9,026). We assessed LTC disparities related to five sociodemographic factors (age, gender, living status, urbanization, income status) and seven types of LTC services. Older adults who lived in a city were more likely to use multiple HCBS and transportation services. Older adults who lived alone were more likely to use home care and meal services but not other types of LTC services. All disparities in service use increased from TLTCP 1.0 to TLTCP 2.0. Part of a symposium sponsored by International Comparisons of Healthy Aging Interest Group.

